# Supplementary material for: 2-Methoxyestradiol Inhibits Radiation-Induced Skin Injuries
Source: Int J Mol Sci. 2022 Apr 10;23(8):4171. doi: 10.3390/ijms23084171 (PMC9032705; doi:10.3390/ijms23084171)
Supplement: Supplementary file 1 [file ijms-23-04171-s001.zip › ijms-1659473-supplementary.pdf]

Supplementary Figure S1

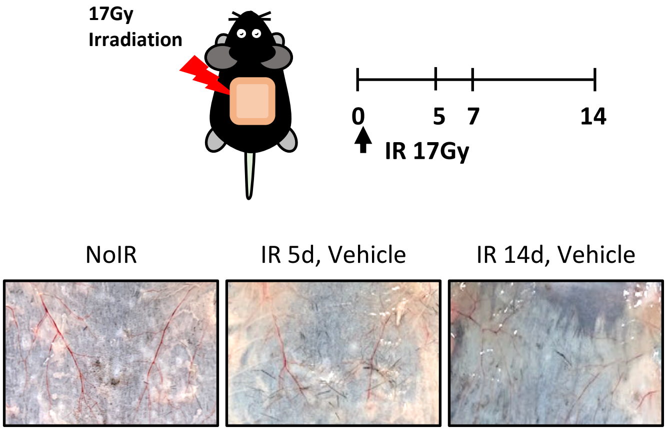

Supplementary Figure S1. Representative images of radiation-induced vascular damage. C57BL/6 mice skin were irradiated with 17 Gy using a 4-cm field. Representative images of 5 days and 2 weeks after irradiation of skin vessels.

Supplementary Figure S2

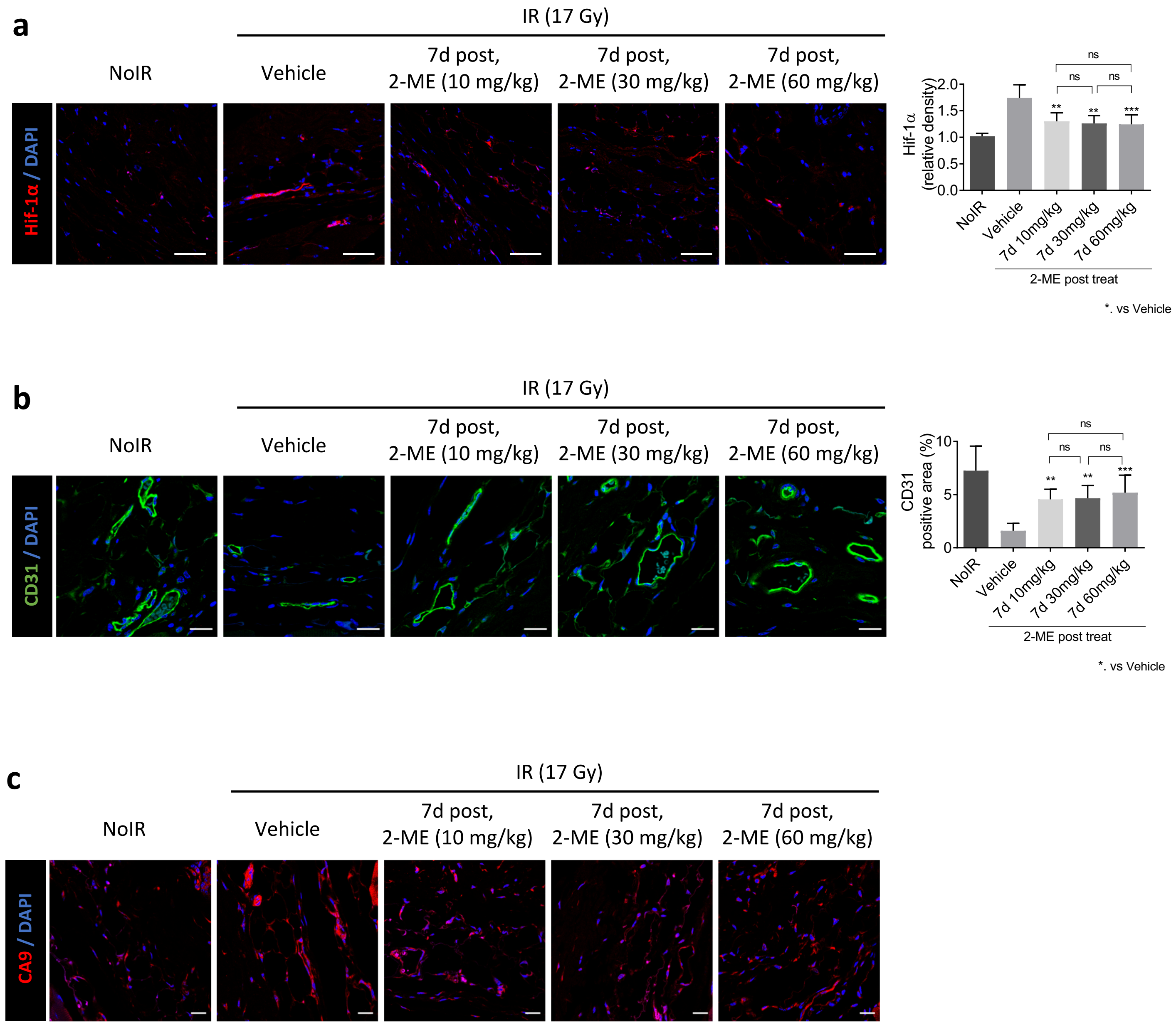

Supplementary Figure S2. 2-ME inhibits irradiation-induced skin vascular CD31, HIF-1α expression, and CA9 expression. C57BL/6 mice skin were irradiated with 17 Gy using a 4-cm field. Mice were orally injected with 2-ME (30 mg/kg) 7 days after irradiation for a total of six times in 3 weeks. (a) Immunofluorescence staining of HIF-1a (Red) in skin tissues from mice that were non-irradiated and irradiated with or without 2-ME treatment. Scale bar=50 μm. Quantification of the HIF-1α$^+$ relative density as an average of five fields (magnification, 200×, n > 5). (b) Immunofluorescence staining of CD31 (green) in skin tissues from mice that were non-irradiated and irradiated with or without 2-ME treatment. Scale bar=20 μm. Quantification of the CD31$^+$ area as an average of five fields (magnification, 200×, n > 5). (c) Immunofluorescence staining of CA9 (Red) in skin tissues from mice that were non-irradiated and irradiated with or without 2-ME treatment. Scale bar=20 μm. Quantification of the CA9$^+$ area per field as an average of five fields (magnification, 200×, n > 5). For all graphs, error bars indicate the SD from n>3 biologically independent experiments (one-way ANOVA for multiple comparisons). * $p < 0.05$, ** $p < 0.01$, *** $p < 0.001$, and **** $p < 0.0001$.

Supplementary Figure S3

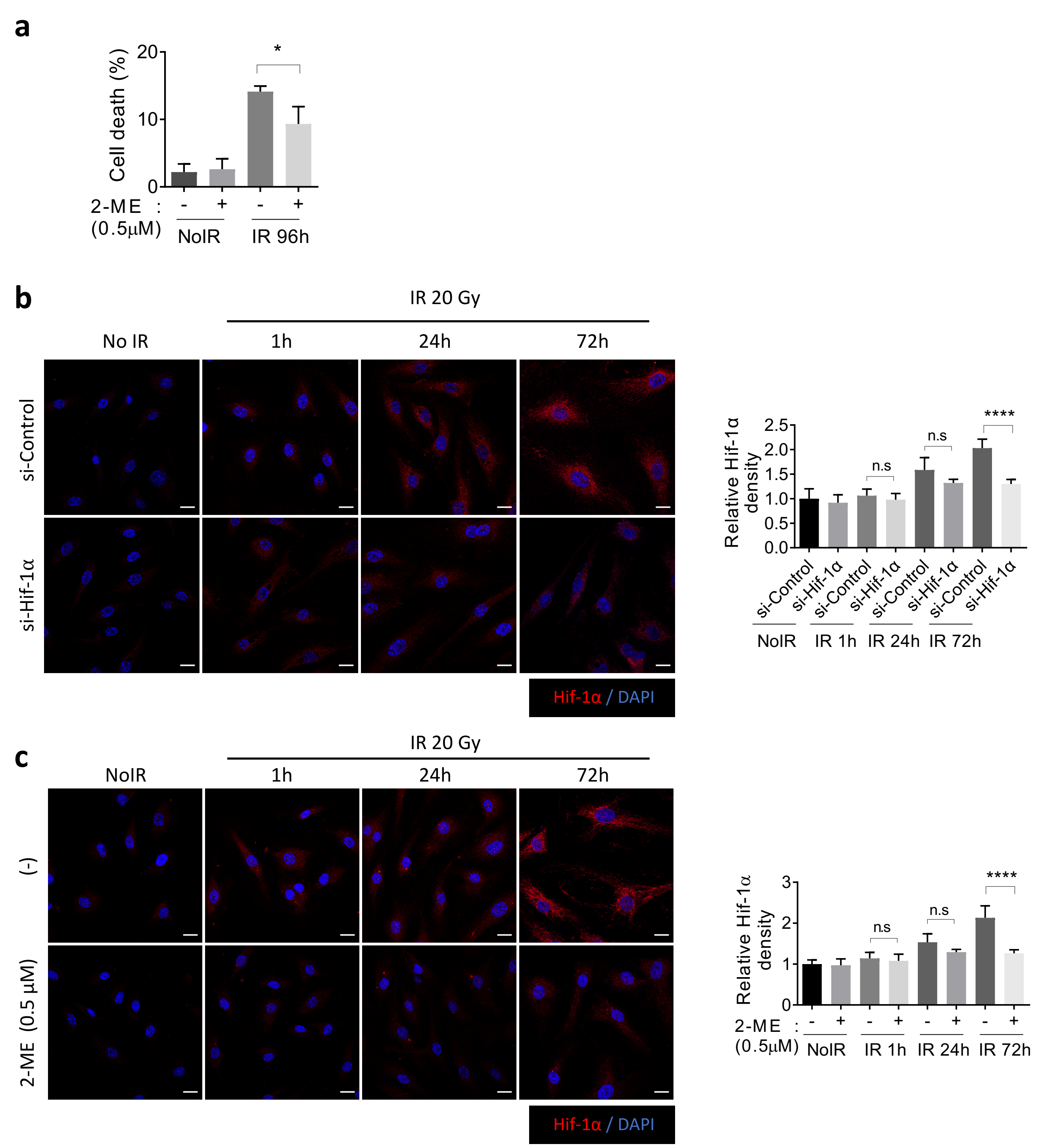

Supplementary Figure S3. 2-ME inhibits radiation-induced cell death and upregulated expression of HIF-1a in HDMECs. (a) The cell death rate in HDMECs 96 h after irradiation and non-irradiation with or without 2-ME (0.5μM) pretreatment determined via FACS analysis using PI staining (n=4 independent experiments). (b) Immunofluorescence staining of HIF-1a in HDMECs transfected with HIF-1a siRNA and control siRNA. Transfected HDMECs were irradiated with 20 Gy and harvested at 1 h, 24 h, and 72 h. Scale bar=20 μm. Bar graph indicates relative density of HIF-1a from five fields (magnification, 200×, n=4 independent experiments) and error bars represent mean ± SD (one-way ANOVA for multiple comparisons). (c) Immunofluorescence staining of HIF-1a in HDMECs treated with 2-ME (0.5 μM) before being irradiated with 20 Gy. The cells were harvested at 1 h, 24 h, and 72 h. Scale bar=20 μM. Bar graph indicates relative density of HIF-1a from five fields (magnification, 200×, n=4 independent experiments) and error bars represent mean ± SD (one-way ANOVA for multiple comparisons). * $p < 0.05$, ** $p < 0.01$, *** $p < 0.001$ and **** $p < 0.0001$.

Supplementary Figure S4

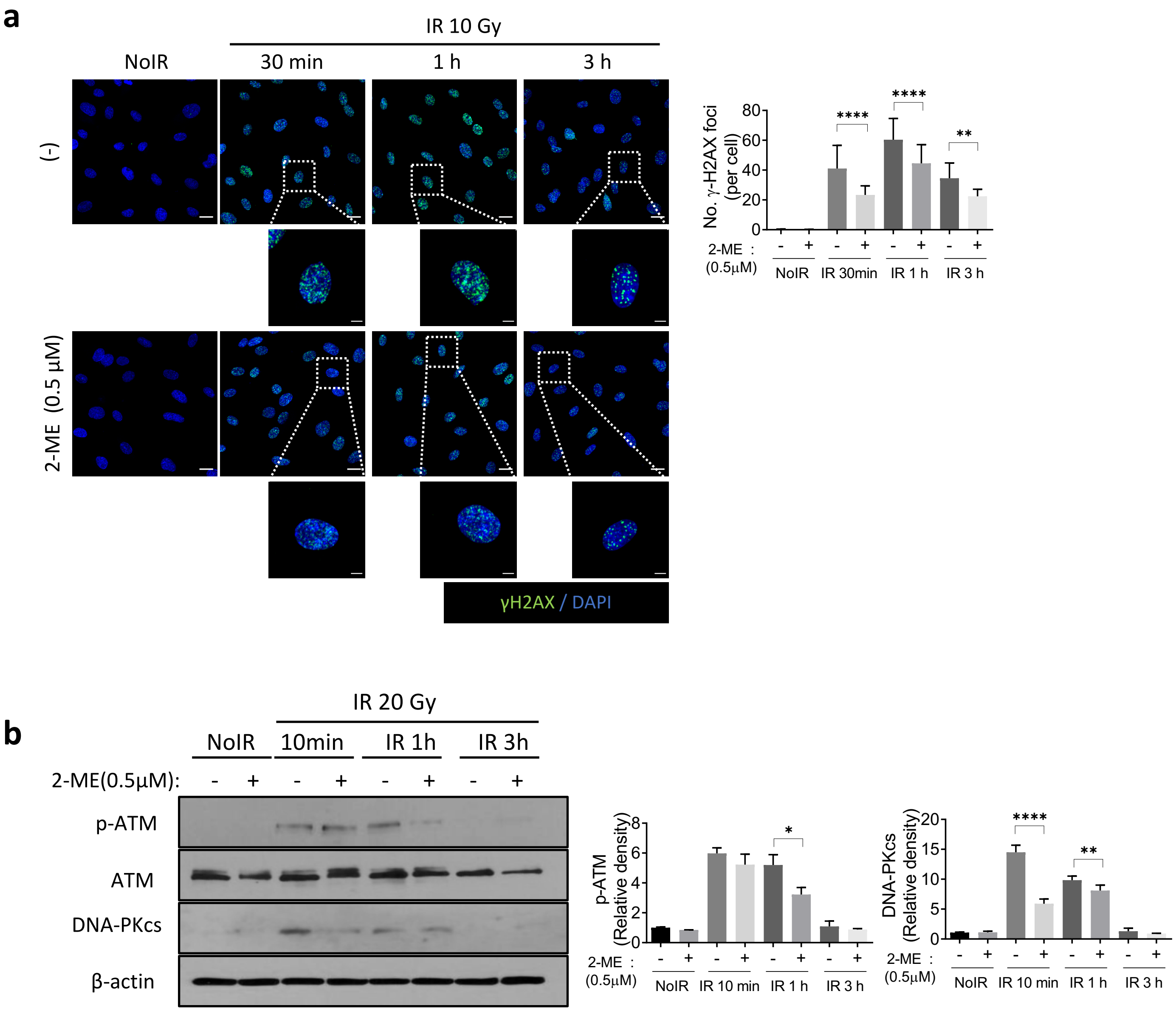

Supplementary Figure S4. 2-ME inhibits radiation-induced DNA damage in HUVECs and protein expression in HDMECs. (a) Immunofluorescence staining γ-H2AX in HUVECs treated with 2-ME (0.5 µM) before 30 min irradiated with 10 Gy. The cells were harvested at 30 min, 1 hr, and 3 hr. Scale bar=20 µm; scale bar of the cropped images=5 µm. Bar graphs indicate the number of γ-H2AX foci per cell. The average numbers of foci/cell were each determined from five fields (magnification, 200×). (b) HDMECs were irradiated with 20 Gy after treated with 2-ME (0.5 µM) and harvest at 10 min, 1 hr, and 3hr. DNA damage repair protein level, p-ATM, ATM, DNA-PKcs, and β-actin were assessed by Western blotting. For all graphs, error bars indicate the SD from n>3 biologically independent experiments (one-way ANOVA for multiple comparisons). * $p < 0.05$, ** $p < 0.01$, *** $p < 0.001$, and **** $p < 0.0001$.
